# A stage specific NETs-related signature in alcoholic steatohepatitis: from molecular subtyping to therapeutic vulnerabilities

**DOI:** 10.3389/fimmu.2025.1711388

**Published:** 2026-01-14

**Authors:** Wei Gao, Zhiyong Lin, Yuntao Bao, Mingjiang Liu, Guangtao Ma, Xianxiang Chen, Shuiping Yu, Yonglian Zeng

**Affiliations:** 1 Division of Hepatobiliary Surgery, The First Affiliated Hospital of Guangxi Medical University, Nanning, China; 2 Key Laboratory of Early Prevention and Treatment for Regional High Frequency Tumor (Guangxi Medical University), Ministry of Education, Nanning, China; 3 Guangxi Key Laboratory of Immunology and Metabolism for Liver Diseases, Guangxi Medical University, Nanning, China

**Keywords:** alcoholic steatohepatitis, CXCL6, FOS, MMP7, neutrophil extracellular traps

## Abstract

**Background:**

Alcohol-associated steatohepatitis (ASH) is a globally prevalent liver disease, with robust evidence implicating neutrophil extracellular traps (NETs) as a central pathological phenomenon driving inflammation and progression. However, the core genomic signatures that govern NETs and underlying molecular mechanisms within the ASH microenvironment remain poorly defined.

**Methods:**

Building on the prominent NETs formation phenomenon in ASH, we established a core pool of NETs-related hub genes through intersection of ASH-derived differentially expressed genes (DEGs), key WGCNA modules, and a curated NETs gene set. From this NET-focused pool, a consensus of three machine learning algorithms (LASSO, SVM, RF) distilled a final diagnostic signature, which was rigorously validated in training and external cohorts via ROC analysis and neural networks. Patient heterogeneity was then investigated using consensus clustering with this signature, followed by immune profiling and functional validation in human and mouse ASH models. Therapeutic potential was explored through drug database enrichment and molecular docking.

**Results:**

A NETs-focused three-gene signature (FOS, MMP7, CXCL6) achieved exceptional diagnostic accuracy for ASH (AUC = 1.00 in training; 0.983 in validation). It stratified ASH into a Metabolic-dominant (C1) subtype and a Pro-inflammatory (C2) subtype, the latter exhibiting higher MMP7/CXCL6, lower FOS, and enriched cytotoxic infiltration. *In vivo*, FOS rose in acute injury but declined in chronic models and human ASH, whereas MMP7/CXCL6 remained elevated, suggesting a temporal shift from acute FOS-dominant response to sustained MMP7/CXCL6-mediated inflammation. Finally, drug-gene interaction analysis identified several potential therapeutic modulators, including N-acetylcysteine (NAC), with predicted high binding affinities to FOS and MMP7.

**Conclusion:**

FOS, MMP7, and CXCL6 constitute a clinically actionable signature capturing the stage-specific dynamics of NETs-driven inflammation in ASH. Beyond its diagnostic and stratifying utility, this signature highlights potential therapeutic avenues for clinical intervention.

## 1 Introduction

The abuse of alcohol has led to a significant increase in patients with alcoholic liver disease (ALD), which is a leading cause of cirrhosis, hospitalizations, and liver transplantation in developed countries (1, 2). This syndrome manifests as a pathological spectrum that extends from simple steatosis to cirrhosis and hepatocellular carcinoma (3, 4). Within this spectrum, ASH represents a distinct, severe inflammatory phenotype characterized by profound hepatocellular injury and a high risk of short-term mortality (5–7). Currently, therapeutic options for severe ASH remain limited, and these therapies provide only limited long-term survival benefits (8, 9). Although liver transplantation serves as a potential cure, its application is constrained by organ availability and concerns relapse. Therefore, effective strategies for early detection and intervention are urgently needed to arrest ASH expansion and progression; however, we still lack effective methods to assess ASH progression.

Neutrophils are central to the immunopathology of ASH. Clinically, this is manifested by marked peripheral neutrophilia and massive neutrophil infiltration within the liver (10). Besides, the extent of hepatic neutrophil accumulation correlates strongly with disease severity indices, including the Model for End-Stage Liver Disease (MELD) score and increased 90-day mortality, establishing it as an independent predictor of poor prognosis (11, 12). Although neutrophils are essential for host defense, their activation becomes maladaptive within the ASH microenvironment, culminating in the explosive release of NETs (13). These web-like DNA-protein complexes, decorated with cytotoxic histones and granule proteases such as neutrophil elastase, directly induce hepatocyte death and amplify local inflammation (14, 15).

In ASH, the molecular machinery driving this NETs-related inflammation is complex and multifactorial. Chronic alcohol exposure creates a state of immune dysregulation, priming neutrophils for enhanced NET formation and oxidative burst while simultaneously impairing their phagocytic capacity, as evidenced by the accumulation of low-density neutrophils (11). This injurious cycle is further compounded by alcohol-induced gut microbiota dysbiosis and intestinal barrier dysfunction. These systemic disruptions facilitate the translocation of pathogen-associated molecular patterns (PAMPs) and damage-associated molecular patterns (DAMPs) into the portal circulation (16). In the liver, PAMPs engage Toll-like receptor 4 (TLR4) and downstream MyD88 signaling on Kupffer cells, while DAMPs activate the NLRP3 inflammasome, triggering the release of IL-1β and IL-18 (16–18). These signals establish a feed-forward loop that intensifies neutrophil recruitment and sustains inflammation. Oxidative stress acts as a critical nexus for NET formation. Reactive Oxygen Species (ROS) generated via NADPH oxidase and mitochondrial dysfunction serve as key triggers for NETs formation (19). At the transcriptional level, this unchecked activation is driven by the loss of intrinsic checkpoints. As reported by Li M et al., miR-223 is pathologically downregulated in neutrophils from both human ASH patients and murine models, resulting in the upregulation of its direct targets, IL-6 and p47^phox^, which removes the “molecular brake” on ROS production and perpetuates spontaneous NET release (20). Additionally, miR-155 promotes alcohol-induced fatty liver inflammation and fibrosis in mice (21).

Despite these mechanistic insights, a critical knowledge gap remains. Most studies have focused on isolated pathways rather than the systemic transcriptomic landscape. Consequently, the core molecular signature that orchestrates neutrophil recruitment and governs the transition from acute activation to sustained NET-driven inflammation is poorly defined. In addition, clinically actionable biomarkers that can capture this specific immunopathology are lacking. To address this, we hypothesized that a specific, robust NETs-related gene signature acts as a central determinant of inflammatory intensity in ASH. In the present study, we integrated multi-omics bioinformatics with machine learning algorithms to distill a core diagnostic signature (FOS, MMP7, CXCL6). We validated the diagnostic performance of this signature in multiple cohorts and, crucially, elucidated its stage-specific dynamics using both human samples and *in vivo* models. Our findings provide a comprehensive framework for understanding NETs-driven inflammation in ASH and highlight novel therapeutic avenues.

## 2 Materials and methods

### 2.1 Data acquisition and clinical sample collection

Public gene expression data were acquired from the Gene Expression Omnibus (GEO) repository (https://www.ncbi.nlm.nih.gov/geo/). The training cohort was established by integrating two microarray datasets, GSE103580 (n=13 ASH samples; GPL13667 platform) and GSE28619 (n=15 ASH samples, n=7 normal controls; GPL570 platform). To eliminate technical variations, batch effects between these datasets were corrected using the “ComBat” algorithm within the “sva” package (v3.56.0) (22), resulting in a unified cohort of 28 ASH and 7 normal samples. The effective removal of batch effects was confirmed by principal component analysis (PCA). For external validation, two high-throughput sequencing datasets, GSE142530 and GSE155907, were similarly merged and batch-corrected, yielding a validation cohort of 15 ASH and 16 normal samples. The study was conducted with approval from the Institutional Ethics Committee of the First Affiliated Hospital of Guangxi Medical University (No. 2025-E0764), adhering to the Declaration of Helsinki. Liver specimens were prospectively collected from ASH patients and normal controls, all of whom provided written informed consent. Upon collection, samples were immediately snap-frozen in liquid nitrogen and stored at –80 °C for subsequent experiments.

### 2.2 Identification of DEGs

Raw expression matrices from the GEO cohorts were processed using the limma package, encompassing background correction, data normalization, and probe-to-symbol mapping. DEGs between ASH and normal samples were identified using the criteria of |log_2_ fold-change| > 1.5 and an adjusted P-value < 0.05. Volcano plots and heatmaps were generated using the ggplot2 and heatmap packages to visualize the DEGs. To elucidate the biological functions of these DEGs, we performed Gene Ontology (GO) and Kyoto Encyclopedia of Genes and Genomes (KEGG) pathway enrichment analyses.

### 2.3 WGCNA network construction and key module identification

To identify clinically relevant gene modules, we performed a weighted gene co-expression network analysis (WGCNA) using the “WGCNA” package (v1.73) (23). After filtering for low-variance genes to enhance network robustness, an optimal soft-thresholding power (β) was selected to satisfy the scale-free topology criterion. The resulting adjacency matrix was transformed into a Topological Overlap Matrix (TOM). Modules were delineated using dynamic tree cutting, and modules with high similarity (dissimilarity threshold < 0.3) were merged. The association between module eigengenes and clinical traits was quantified using Pearson correlation. Modules exhibiting the strongest correlations with ASH were selected for further analysis, and hub genes were identified based on high module membership (MM) and gene significance (GS).

### 2.4 Core gene identification and immune infiltration analysis

To identify core genes, we first compiled an extensive list of NETs-related genes from the GeneCards database (https://www.genecards.org/) (24). This list was then intersected with the previously identified DEGs and key WGCNA module genes to yield a final set of core NETs-associated genes in ASH, with the overlap depicted in a Venn diagram. To characterize these core genes, a protein-protein interaction (PPI) network was generated to investigate their functional relationships. The expression patterns, inter-gene correlations, and genome-wide significance of the 22 identified core genes were visualized using boxplots, a correlation matrix, and a Manhattan plot, respectively. To characterize the immune microenvironment, we estimated the infiltration levels of various immune cell subsets using the single-sample Gene Set Enrichment Analysis algorithm implemented in the “GSEABase” (v1.70.0) (25) and “GSVA” (v2.2.0) (26) packages. The relationships between core genes and immune cell populations were evaluated using Spearman correlation analysis, and the results were presented as a heatmap.

### 2.5 Machine learning-based identification of NETs-related diagnostic biomarkers

To identify a robust set of NETs-related diagnostic biomarkers, we utilized an ensemble strategy based on three distinct machine learning algorithms. Least Absolute Shrinkage and Selection Operator (LASSO) regression was conducted via the “glmnet” package (v4.1.0) (27) to select features with non-zero coefficients. A Support Vector Machine with Recursive Feature Elimination (SVM-RFE) was implemented using the “e1071” package (v1.7.16) (28) with 10-fold cross-validation to further refine the selection. A Random Forest (RF) model was trained using the “randomForest” package (v4.7.1.2) (29), and genes with a mean decrease in Gini (MDG) greater than 1 were retained. The final diagnostic signature was composed of those genes that were commonly identified by all three approaches.

### 2.6 Validation of the diagnostic signatures

First, we analyzed the expression of the biomarkers in the training cohort, using the Mann-Whitney U test to determine the significance of differences between groups. Subsequently, the diagnostic performance of each gene and the combined signature was evaluated using Receiver Operating Characteristic (ROC) analysis (“pROC” package, v1.18.5) (30) and a feed-forward neural network model (“neuralnet” and “NeuralNetTools” packages, v1.44.2 and v1.5.3) (31, 32). To facilitate clinical use, a nomogram was developed as a quantitative diagnostic tool using the rms package, and its predictive accuracy was assessed with a calibration plot. Finally, the diagnostic accuracy of the signature was further validated in the independent, combined GEO cohort (GSE142530 and GSE155907).

### 2.7 Identification of NETs-associated molecular subtypes

To explore patient heterogeneity based on the diagnostic signature, an unsupervised consensus clustering analysis was performed to ASH samples via “ConsensusClusterPlus” package (v1.72.0) (33). This process utilized the k-means algorithm with a Euclidean distance metric and was repeated for 50 iterations (pItem=0.8, pFeature=1, maxK=9). The optimal number of clusters was determined by evaluating the cumulative distribution function (CDF) plot and the relative change in the area under the CDF curve. The resulting subtypes (C1 and C2) were visualized by PCA. We then characterized these subtypes by comparing their immune infiltration profiles and pathway activities via GSVA.

### 2.8 Drug prediction and molecular docking

Potential therapeutic compounds targeting the biomarker signature were identified through enrichment analysis against the Drug Signatures Database (DSigDB, https://dsigdb.tanlab.org/DSigDBv1.0/). A drug–gene interaction network was visualized using Cytoscape. Three-dimensional structures of the protein targets were obtained from the RCSB Protein Data Bank (https://www.rcsb.org/structure), and ligand structures were retrieved from PubChem(https://pubchem.ncbi.nlm.nih.gov/). Molecular docking simulations were performed using the CB-Dock2 platform(https://cadd.labshare.cn/cb-dock2/php/index.php) to predict the binding energies and interaction modes between the compounds and protein targets.

### 2.9 Animal studies

Eight- to ten-week-old female C57BL/6 mice were obtained from the Laboratory Animal Center of Guangxi Medical University. All animal experiments were approved by the Medical Ethics Committee of the First Affiliated Hospital of Guangxi Medical University (No. 2025-E0764). The Lieber-DeCarli liquid diets were sourced from Trophic Animal Feed High-Tech Co. Ltd (Nantong, China). The ethanol liquid diet (Cat. TP 4030D) contained 5% (v/v) ethanol. The isocaloric control liquid diet (Cat. TP 4030C) was formulated by substituting ethanol calories with an equivalent caloric amount of maltodextrin-rich dry powder base. Two distinct ASH models were established based on the National Institute on Alcohol Abuse and Alcoholism (NIAAA) protocol (34). For the short-term binge model, mice were acclimated with a control Lieber-DeCarli liquid diet for 5 days. The ASH group then received a 5% (v/v) ethanol diet for 10 days, while the control group remained on an isocaloric diet. On day 16, mice received a single oral gavage of ethanol (31.5% v/v, 5 g/kg body weight) between 7:00 and 9:00 AM. For the chronic model, following acclimation, the ASH group was fed a 5% ethanol diet for 5 weeks, concluding with three consecutive daily gavages of ethanol (31.5% v/v, 5 g/kg body weight) between 7:00 and 9:00 AM. The control group received an isocaloric diet and maltodextrin gavage (9g/kg body weight). we implemented a strict pair-feeding protocol. The daily food intake of the ethanol-fed mice was measured daily at 5:00 PM. Accordingly, the control group was provided with an isocaloric amount of the control diet (TP 4030C) matched to the volume consumed by their ethanol-fed counterparts on the previous day. Control mice also received isocaloric maltodextrin gavages at the corresponding time points. For both models, mice were euthanized 9 hours after the final gavage via CO_2_ inhalation using a gradual-fill method, and death was confirmed by cervical dislocation as a secondary physical method.

### 2.10 Histopathology and biochemical analysis

For histopathological and biochemical evaluation, liver tissues were subjected to several procedures. For the assessment of lipid accumulation, tissues were cryosectioned (10 µm) and subsequently stained with Oil Red O. For morphological analysis, tissues were fixed in formalin, embedded in paraffin, cut into 4 µm sections, and stained with Hematoxylin and Eosin (H&E). In parallel, an automated biochemical analyzer was used to measure the plasma concentrations of alanine aminotransferase (ALT) and aspartate aminotransferase (AST).

### 2.11 Analysis of hepatic oxidative stress and lipid content

Liver tissue homogenates were prepared to measure the levels of superoxide dismutase (SOD), glutathione (GSH), malondialdehyde (MDA), total cholesterol (TC), and triglycerides (TG). All assays were performed using commercial kits (Nanjing Jiancheng Bioengineering Institute, Cat. Nos. A001–3, A006–2, A003–1, A111–1, A110–1) according to the manufacturer’s instructions.

### 2.12 Enzyme-linked immunosorbent assay

The concentrations of TNF-α (NeoBioscience, EMC102a.96), IL-6 (NeoBioscience, EMC004.96), and IL-1β (NeoBioscience, EMC001b.96) in liver homogenates were quantified by ELISA following the manufacturer’s protocols.

### 2.13 Immunohistochemistry

liver sections were deparaffinized and rehydrated. Endogenous peroxidase activity was quenched, and antigen retrieval was performed using citrate (pH 6.0) or EDTA (pH 9.0) buffer. Sections were incubated overnight at 4 °C with primary antibodies against MPO (1:8000, Abcam, ab188211), F4/80 (1:500, CST, 70076S), MMP7 (1:200, Proteintech, 10374-2-AP), or CXCL6 (1:200, Abclonal, TD13470). After incubation with a biotinylated secondary antibody (ZSGB-Bio), signals were developed using DAB and counterstained with hematoxylin. Staining was quantified using ImageJ software (NIH, USA) with color deconvolution. Positive staining was expressed as the percentage of the DAB-positive area relative to the total tissue area.

### 2.14 Immunofluorescence staining

sections underwent deparaffinization, rehydration, and antigen retrieval (EDTA buffer, pH 9.0). For single staining, sections were permeabilized, blocked, and incubated overnight at 4 °C with anti-FOS primary antibody (1:100, Abclonal, A24620), followed by an Alexa Fluor 488–conjugated secondary antibody (1:1000, Proteintech, RGAR002). For double staining of NETs, sections were sequentially incubated with anti-MPO (1:5000, Abcam, ab188211) and anti-Histone H3 (1:100, Abcam, ab281584) antibodies, using a tyramide signal amplification (TSA) kit (ABclonal, RK05902) for signal development with distinct fluorophores. Nuclei were counterstained with DAPI. Images were captured via fluorescence microscopy, and relative expression was quantified in ImageJ as the ratio of the target mean fluorescence intensity to the DAPI intensity.

### 2.15 Western blotting

Total protein was extracted from whole liver tissues using RIPA buffer, and concentrations were determined by a Bradford assay. Equal protein amounts were resolved by SDS-PAGE and transferred to PVDF membranes. Membranes were blocked and incubated overnight at 4 °C with primary antibodies against FOS (1:2000, Abclonal, A24620), MMP7 (1:1000, Proteintech, 10374-2-AP), CXCL6 (1:1000, Abclonal, TD13470), PPAR-α (1:500, Bioss, bs-23398R), PPAR-γ (1:1000, Proteintech, 16643-1-AP), HO-1 (1:1000, Abmart, PY5393S), MDA (1:1000, abcam, ab27642), Arg1 (1:5000, Proteintech, 16001-1-AP), MCP-1 (1:500, CST, 2029S), IL-10 (1:1000, Proteintech, 82191-3-RR) and β-actin (1:10,000, Proteintech, 20536-1-AP). Following incubation with HRP-conjugated secondary antibodies (1:8,000, Proteintech, SA00001-2), protein bands were visualized using a LI-COR imaging system.

### 2.16 Statistical analysis

All statistical analyses were conducted using R software (v4.5.0), IBM SPSS (v26.0), or GraphPad Prism (v10.4.2). Comparisons between two groups were performed with two-tailed unpaired Student’s t-tests or Mann–Whitney U tests. For comparisons involving more than two groups, one-way ANOVA was used. Correlations were assessed using Pearson’s r or Spearman’s ρ coefficients. All experiments were performed with at least three independent biological replicates. A *p*-value < 0.05 was considered statistically significant (*p<0.05; **p<0.01; ***p<0.001).

## 3 Results

### 3.1 Identification of core NETs-related hub genes in ASH

**Figure 1:**
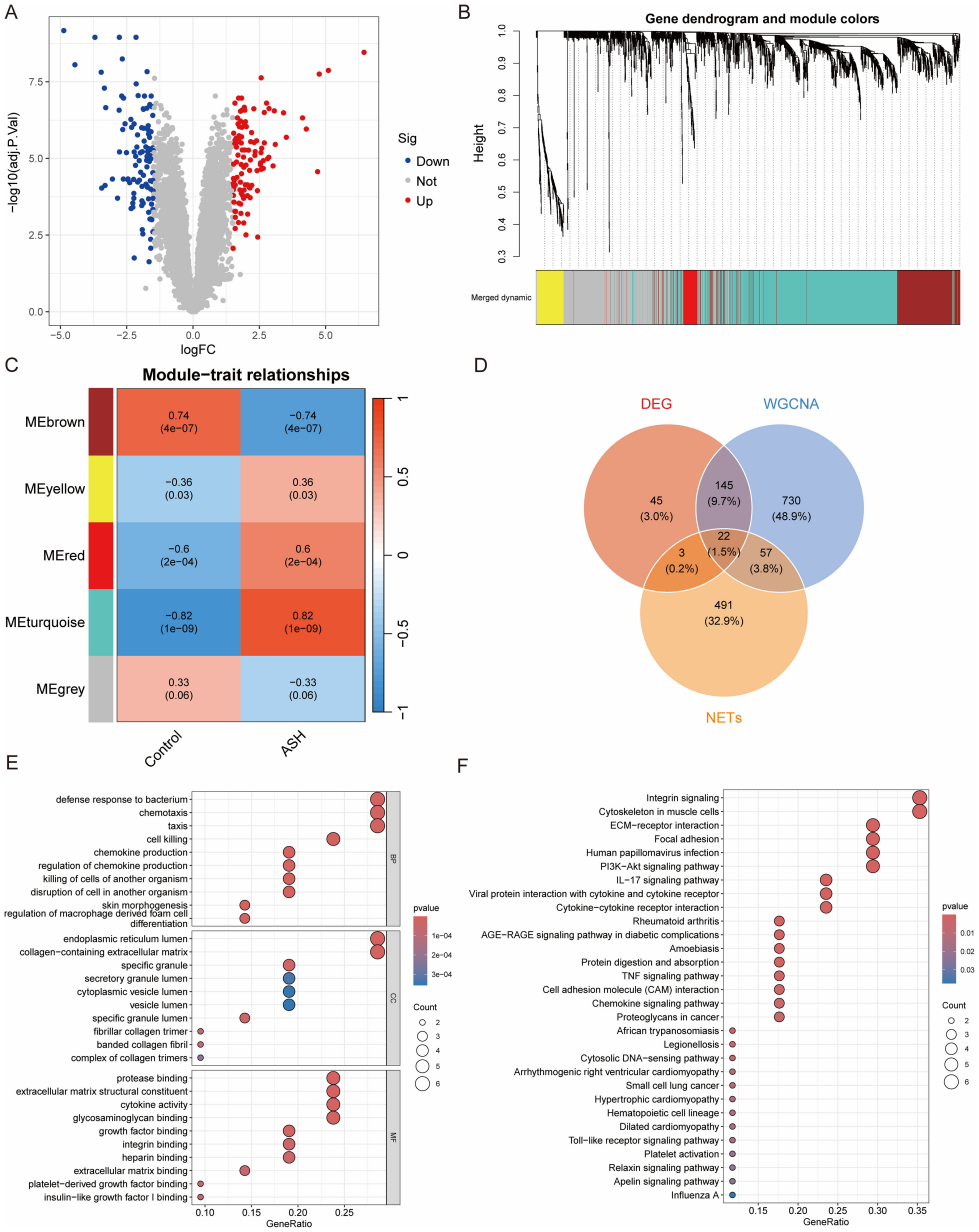
Identification and immune profiling of 22 core hub genes in ASH. **(A)** Volcano plot illustrating differentially expressed genes (DEGs) between ASH and control samples. **(B)** Gene clustering dendrogram, where distinct branches represent co-expression modules, indicated by different colors. **(C)** Heatmap of module-trait relationships, displaying the Pearson correlation coefficient between each module eigengene and the clinical trait. **(D)** Venn diagram showing the overlap between DEGs, the key turquoise WGCNA module, and a curated set of NETs-related genes. **(E, F)** Functional enrichment analysis. Bubble plots depict the top enriched terms from GO biological processes (H) and KEGG pathways (I) for the hub genes.

To systematically explore the molecular landscape of ASH, we analyzed the transcriptome datasets corrected for batch effects (**Supplementary Figure S1A**), identifying a total of 215 DEGs between ASH and control samples (**Figure 1A**). Moving beyond individual gene analysis to capture the systemic transcriptional architecture, we performed a WGCNA. Following the detection and removal of outlier samples (**Supplementary Figure S1B**), a soft-thresholding power (β) of 13 was chosen to satisfy a scale-free topology fit (**Supplementary Figure S1C**), resulting in the identification of five distinct gene modules (**Figure 1B**). Notably, the turquoise module showed the most significant correlation with ASH clinical status (**Figure 1C**), with this association further supported by its high overall gene significance (**Supplementary Figure S1D**). Given the established role of neutrophils and NETs in ASH, we are next integrated the 215 DEGs, genes within the significant turquoise module, and a curated set of NETs-related genes yielded a core set of 22 hub genes (**Figure 1D**). These genes were not only differentially expressed in ASH versus control samples but also demonstrated strong co-expression patterns and dense functional connections, by correlation analysis and a PPI network, respectively (**Supplementary Figures S2A–C**).

Further functional enrichment analysis of the 22 hub genes elucidated a profound signature of neutrophil-mediated immunity. GO enrichment analysis was significantly enriched for biological processes such as “defense response to bacterium” and “chemotaxis”, cellular components specific to neutrophils such as the “secretory granule lumen”, and molecular functions including “cytokine activity” (**Figure 1E**). This was corroborated by KEGG analysis, which implicated key inflammatory cascades, including the IL-17, Cytokine-cytokine receptor interaction and TNF signaling pathways (**Figure 1F**). Furthermore, analysis of immune cell infiltration demonstrated that the expression of hub genes was strongly correlated with the abundance of multiple immune cell populations, underscoring their potential role in modulating the hepatic immune microenvironment in ASH (**Supplementary Figures S2D, E**). In summary, we identified 22 core NETs-related hub genes in ASH that are closely associated with a neutrophil-driven inflammatory microenvironment.

### 3.2 Machine learning algorithms construct core biomarkers for ASH diagnosis

**Figure 2:**
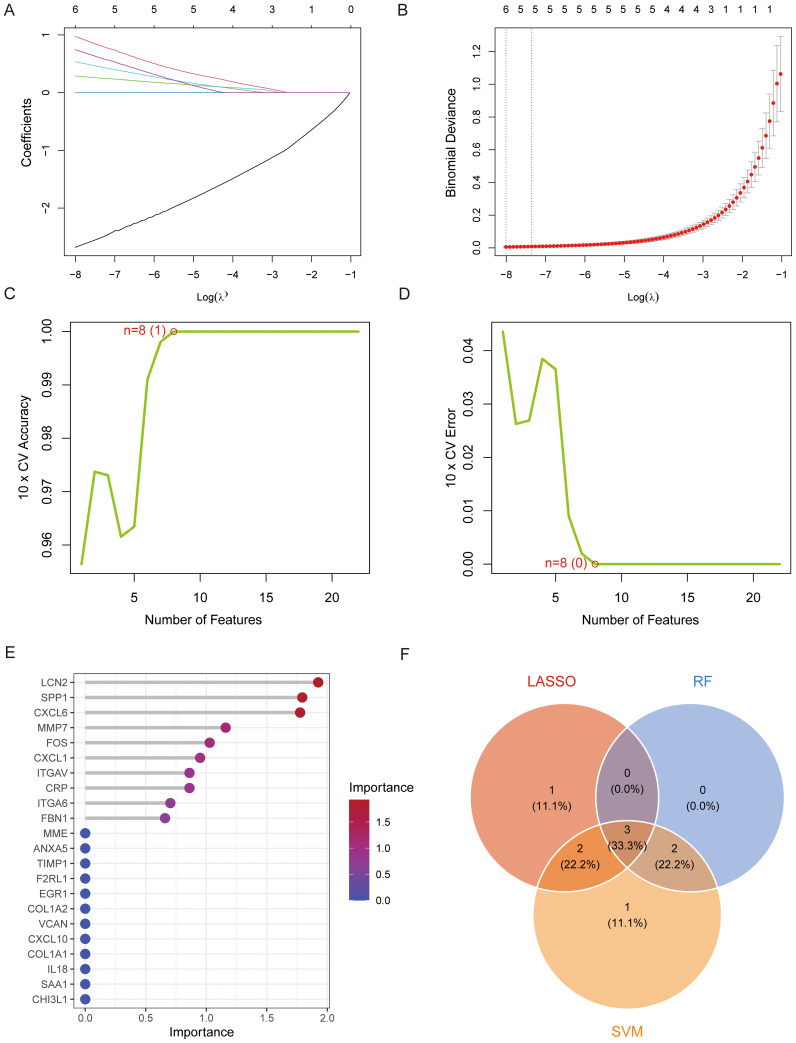
Machine learning-based selection of NETs-related diagnostic biomarkers for ASH. **(A, B)** Feature selection using the LASSO regression model. **(A)** Regularization paths of the hub gene coefficients. **(B)** Determination of the optimal lambda value via a 10-fold cross-validation scheme. **(C, D)** Performance of the SVM-RFE model in identifying the optimal number of features. The plots depict **(C)** the model’s accuracy and **(D)** its error rate, each plotted against the number of features. **(E)** Feature importance plot from the Random Forest model, ranked by mean decrease in Gini index. **(F)** Venn diagram showing the intersection of features selected by LASSO, SVM-RFE, and RF, which yielded three final diagnostic biomarkers.

To construct stable and parsimonious diagnostic biomarkers for ASH, we employed a machine learning approach to screen core candidate factors from 22 core NETs-related hub genes. LASSO regression analysis identified 6 genes with non-zero coefficients at the optimal lambda value (**Figures 2A, B**). The SVM-RFE method identified 8 key feature genes (**Figures 2C, D**). Meanwhile, a Random Forest model ranked genes by importance, and the top 5 were selected (**Figure 2E**). The convergence of these three distinct algorithms, visualized in a Venn diagram, identified a final, high-confidence signature of three genes: FOS, MMP7, and CXCL6 (**Figure 2F**).

### 3.3 Diagnostic performance and external validation of NETs-related biomarkers for ASH

**Figure 3:**
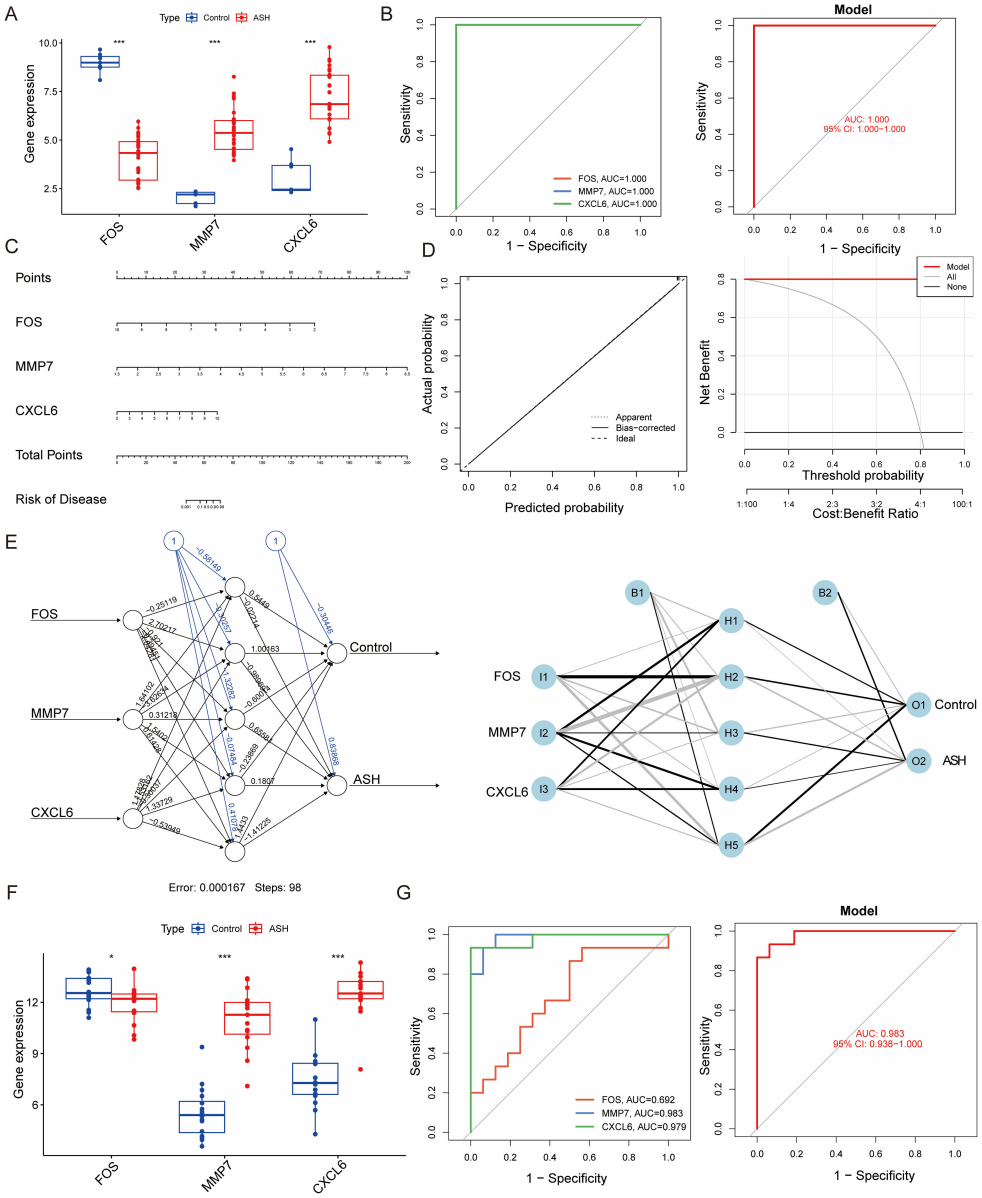
Construction and performance evaluation of a three-gene diagnostic signature for ASH. **(A)** Expression levels of FOS, MMP7, and CXCL6 within the training cohorts (integrated from microarray datasets GSE103580 and GSE28619 following batch effect correction). **(B)** ROC analysis was used to evaluate the diagnostic performance of three-gene signature in the training cohort. **(C)** A nomogram integrating the three biomarkers to predict the probability of ASH. **(D)** Calibration curve assessing the predictive accuracy of the nomogram and a decision curve analysis evaluating its clinical utility. **(E)** Architecture and weights of a neural network model, illustrating the contribution of each gene to ASH prediction. **(F)** Expression levels of the three genes in the external validation cohort (integrated from RNA-seq datasets GSE142530 and GSE155907 after batch correction). **(G)** ROC analysis of the individual genes and the combined model in the external validation cohort (AUC = 0.983). *p < 0.05, ***p < 0.001.

We next systematically evaluated the diagnostic efficacy of the identified signature. In the training cohort, ASH patients exhibited significantly upregulated expression of MMP7 and CXCL6, but downregulated expression of FOS compared to controls (**Figure 3A**). ROC curve analysis demonstrated high discriminatory power, with each gene individually achieving an AUC of 1.000 (**Figure 3B**). To enhance clinical applicability, we developed a nomogram integrating the three biomarkers for individualized risk prediction (**Figure 3C**). The calibration plot demonstrated a high degree of consistency between the predicted probabilities and the actual outcomes, while the Decision Curve Analysis indicated that the model offered a positive net benefit across a broad range of threshold probabilities (**Figure 3D**). A concurrently trained neural network model also showed rapid convergence and minimal error, further supporting the signature’s predictive power (**Figure 3E**). Crucially, to address potential overfitting and ensure generalizability, we validated these findings in an independent external cohort. The expression patterns of the three markers were consistent with the training set (**Figure 3F**). Most importantly, the combined three-gene signature achieved an outstanding AUC of 0.983 in this validation set, confirming its robust and generalizable diagnostic accuracy (**Figure 3G**).

### 3.4 NETs-related signature uncovers two distinct molecular subtypes of ASH

**Figure 4:**
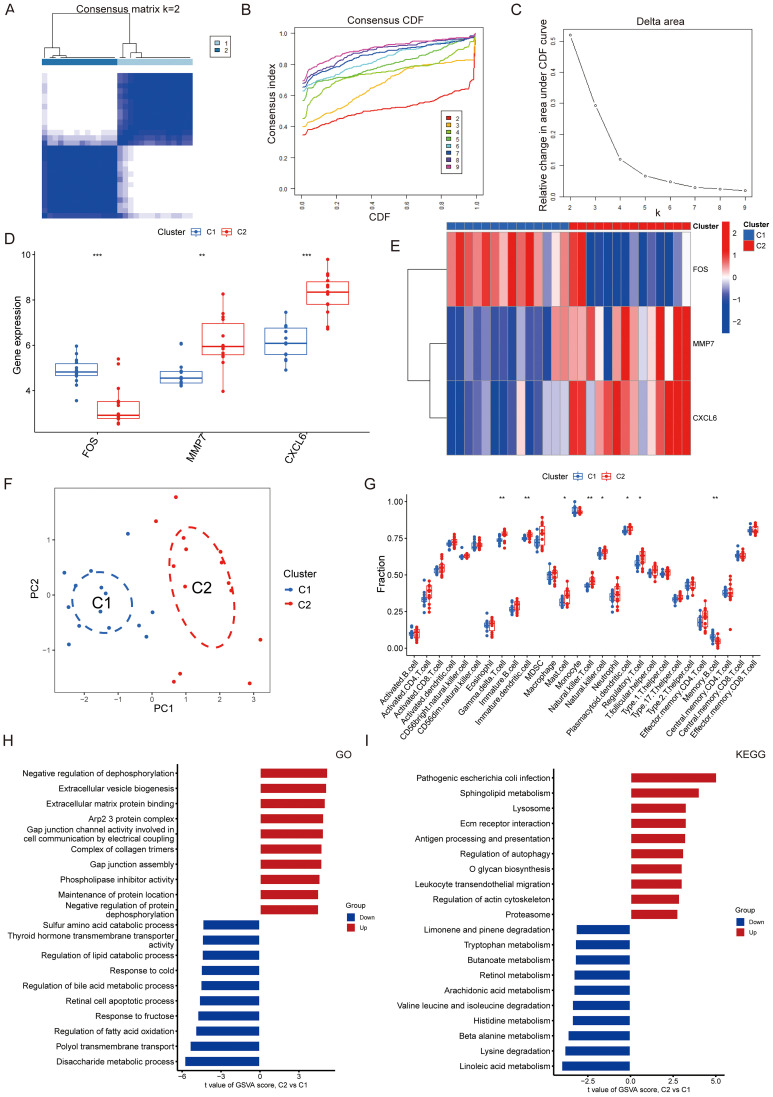
Identification and characterization of NETs-related molecular subtypes in ASH. **(A–C)** The optimal cluster number was identified as k=2 through unsupervised consensus clustering. This conclusion is supported by evidence from the consensus matrix at k=2 **(A)**, the CDF plots for k values from 2 to 9 **(B)**, and the delta area plot **(C)**. **(D)** Box plots showing the expression levels of FOS, MMP7, and CXCL6 in clusters C1 and C2. **(E)** Heatmap showing distinct expression profiles of representative genes between the two subtypes. **(F)** PCA plot visualizing the separation of samples belonging to the two molecular subtypes. **(G)** Differential immune cell infiltration profiles between the two clusters. **(H, I)** GSVA results revealing significantly enriched GO biological processes **(H)** and KEGG pathways **(I)** for each subtype.

To determine whether our three-gene signature could reveal molecular heterogeneity within the ASH patient cohort, we performed unsupervised consensus clustering. This analysis identified two distinct subtypes, designated Cluster 1 (C1) and Cluster 2 (C2), as the optimal classification (**Figures 4A–C**). PCA analysis visualization showed clear separation between the two subtypes. C2 was characterized by higher MMP7 and CXCL6 but lower FOS expression relative to C1 (**Figures 4D–F**). This finding is highly consistent with our predictive model data, suggesting that the ASH diagnostic model we constructed is closely associated with patient stratification.

We next analyzed the gene functions and immune characteristics of these two types of patients. Immunophenotyping analysis revealed marked differences between the two subtypes. The C2 subtype was characterized by a proinflammatory and immunologically active microenvironment, enriched with activated γδ T cells, NK cells, and CD8+ T cells. whereas C1 was characterized by a greater abundance of memory B cells (**Figure 4G**). GSVA analysis further confirmed these distinctions. C2 was enriched for immune and stromal pathways, including lysosome, autophagy, and ECM-receptor interaction, indicative of active inflammation and tissue remodeling. In contrast, C1 displayed a predominantly metabolic phenotype, with enrichment in amino acid, lipid, and bile acid metabolism pathways (**Figures 4H, I**). These findings demonstrate that our NETs-related signature can effectively stratify ASH patients into subgroups with distinct underlying pathobiological mechanisms: a pro-inflammatory, immune-active subtype (C2) and a subtype defined by a predominantly metabolic dysregulation (C1).

### 3.5 Experimental and clinical validation of the NETs-related signature expression

**Figure 5:**
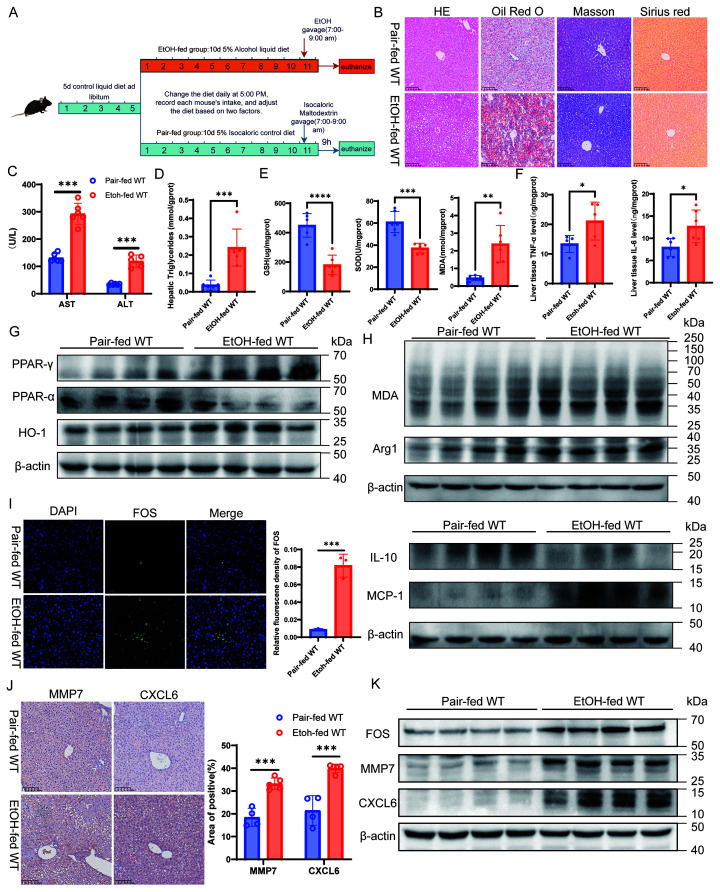
Validation of the NETs signature in a short-term NIAAA mouse model of ASH. **(A)** Schematic representation of the experimental design for the short-term NIAAA model. **(B)** Representative liver histology showing H&E, Oil Red O, Masson’s trichrome, and Sirius red staining (200× magnification; scale bar = 100 μm). **(C)** Serum levels of aspartate aminotransferase (AST) and alanine aminotransferase (ALT). **(D)** Quantification of hepatic triglyceride (TG) content. **(E)** Assessment of hepatic oxidative stress levels indicated by glutathione (GSH), superoxide dismutase (SOD) activity, and malondialdehyde (MDA) content. **(F)** ELISA analysis of hepatic pro-inflammatory cytokines TNF-α and IL-6 levels. **(G)**Western blot analysis of lipid metabolism-related proteins (PPAR-γ, PPAR-α) and the antioxidant enzyme HO-1 in whole liver lysates. **(H)** Western blot analysis of oxidative stress and inflammation markers (MDA, Arg1, MCP-1, and IL-10) in whole liver lysates. **(I)** Immunofluorescence staining of FOS **(H)** (400×; scale bar = 50 μm) and quantification of the relative fluorescence intensity of FOS. **(J)** Immunohistochemical staining of MMP7 and CXCL6 **(G)** (200×; scale bar = 100 μm) and quantification of the positive area. **(K)** Western blot analysis of FOS, MMP7, and CXCL6 protein expression in whole liver lysates. Data are presented as mean ± SEM. *p < 0.05, **p < 0.01, ***p < 0.001..

To validate our bioinformatic findings and explore the underlying mechanisms, we first utilized a short-term NIAAA model (**Figure 5A**). This model successfully recapitulated characteristic ASH features, including steatosis, liver damage, oxidative stress (**Figures 5B–E**; **Supplementary Figure S3A**), and significant inflammation and immune infiltration (**Figure 5F**; **Supplementary Figures S3B, C**). We also confirmed enhanced NETs formation (**Supplementary Figure S3D**). Notably, collagen deposition and fibrosis were not yet observed at this early stage (**Figure 5B**). Western blot analysis of whole liver tissue lysates revealed alterations in metabolic and immune regulators associated with these pathological changes. We observed a downregulation of PPAR-α alongside an upregulation of PPAR-γ (**Figure 5G**). Given the absence of fibrosis, this PPAR-γ activation was accompanied by elevated Arg1 levels (**Figure 5H**), likely reflecting an initial compensatory M2-like reparative response. Nevertheless, this response was insufficient to mitigate injury, as evidenced by profound oxidative stress (elevated HO-1 and MDA) and a dysregulated inflammatory milieu (MCP-1 upregulation, IL-10 suppression) (**Figures 5G, H**). Consistent with our transcriptomic data, MMP7 and CXCL6 were significantly upregulated. Interestingly, we observed a marked upregulation of FOS protein expression (**Figures 5I–K**), which diverged from our observations in the chronic human ASH cohort.

**Figure 6:**
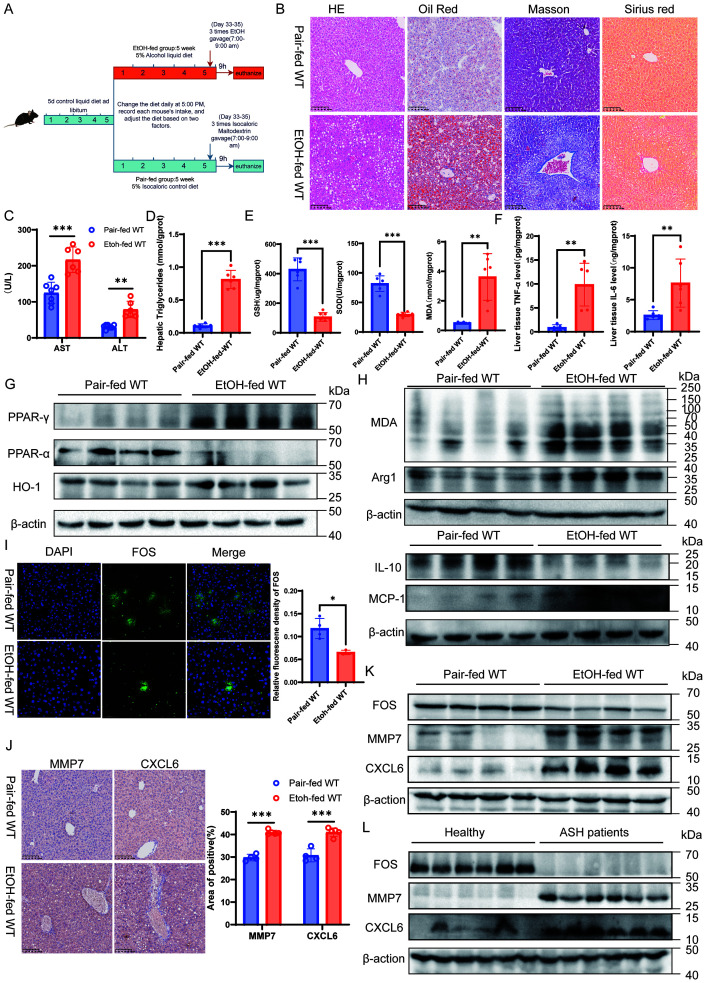
Validation of the NETs signature in a chronic ASH mouse model and in ASH patients. **(A)** Flowchart illustrating the protocol for the chronic-plus-binge ethanol feeding model. **(B)** Representative liver histopathological images stained with H&E, Oil Red O, Masson’s trichrome, and Sirius Red (200× magnification; scale bar = 100 μm). **(C)** Serum AST and ALT levels. **(D)** Hepatic TG and TC content. **(E)** Analysis of hepatic oxidative stress markers (GSH, SOD, MDA). **(F)** Hepatic levels of pro-inflammatory cytokines TNF-α, IL-6. **(G)** Western blot analysis of PPAR-γ, PPAR-α, and HO-1 protein expression in whole liver lysates. **(H)** Western blot analysis of MDA, Arg1, MCP-1, and IL-10 protein levels in whole liver lysates. **(I)** Immunofluorescence detection of FOS and quantification of FOS relative fluorescence intensity (400×; scale bar =50 μm). **(J)** Immunohistochemical staining of MMP7 and CXCL6 alongside quantitative analysis of the positive staining area (200×; scale bar = 100 μm). **(K)** Western blot analysis of FOS, MMP7, and CXCL6 protein expression in the chronic mouse model using whole liver lysates. **(L)** Western blot analysis of FOS, MMP7, and CXCL6 protein expression in whole liver lysates from healthy and ASH patients. Data are presented as mean ± SEM. *p < 0.05, **p < 0.01, ***p < 0.001.

We next employed a long-term NIAAA model to represent a more established chronic disease state (**Figure 6A**). This model maintained a persistent ASH phenotype, with sustained lipid accumulation, inflammation (**Figures 6B–F**; **Supplementary Figures S4A–C**), and consistent NETs deposition (**Supplementary Figure S4D**), but crucially exhibited clear signs of fibrosis initiation (**Figure 6B**). The dysregulation of the metabolic, oxidative, and inflammatory milieus persisted (**Figures 6G, H**). Notably, in this chronic setting, the role of Arg1 appeared to evolve, coinciding with collagen deposition (**Figure 6H**), suggesting a shift from repair to fibrogenesis.While MMP7 and CXCL6 expression remained consistently elevated (**Figures 6J–L**), FOS expression displayed a temporal divergence. In stark contrast to the short-term model, FOS protein levels were now significantly downregulated in the livers of these mice (**Figures 6I, K**). This downregulation precisely mirrored the expression pattern observed in our human ASH patient samples (**Figure 6L**). These findings confirm that MMP7 and CXCL6 are stably upregulated markers of alcoholic liver injury. More importantly, they uncover a dynamic, duration-dependent regulatory pattern for FOS, which suggests that while FOS is induced during acute stress, its expression is exhausted or suppressed during chronic disease progression.

### 3.6 Drug enrichment and molecular docking targeting the NETs-related signature

**Figure 7:**
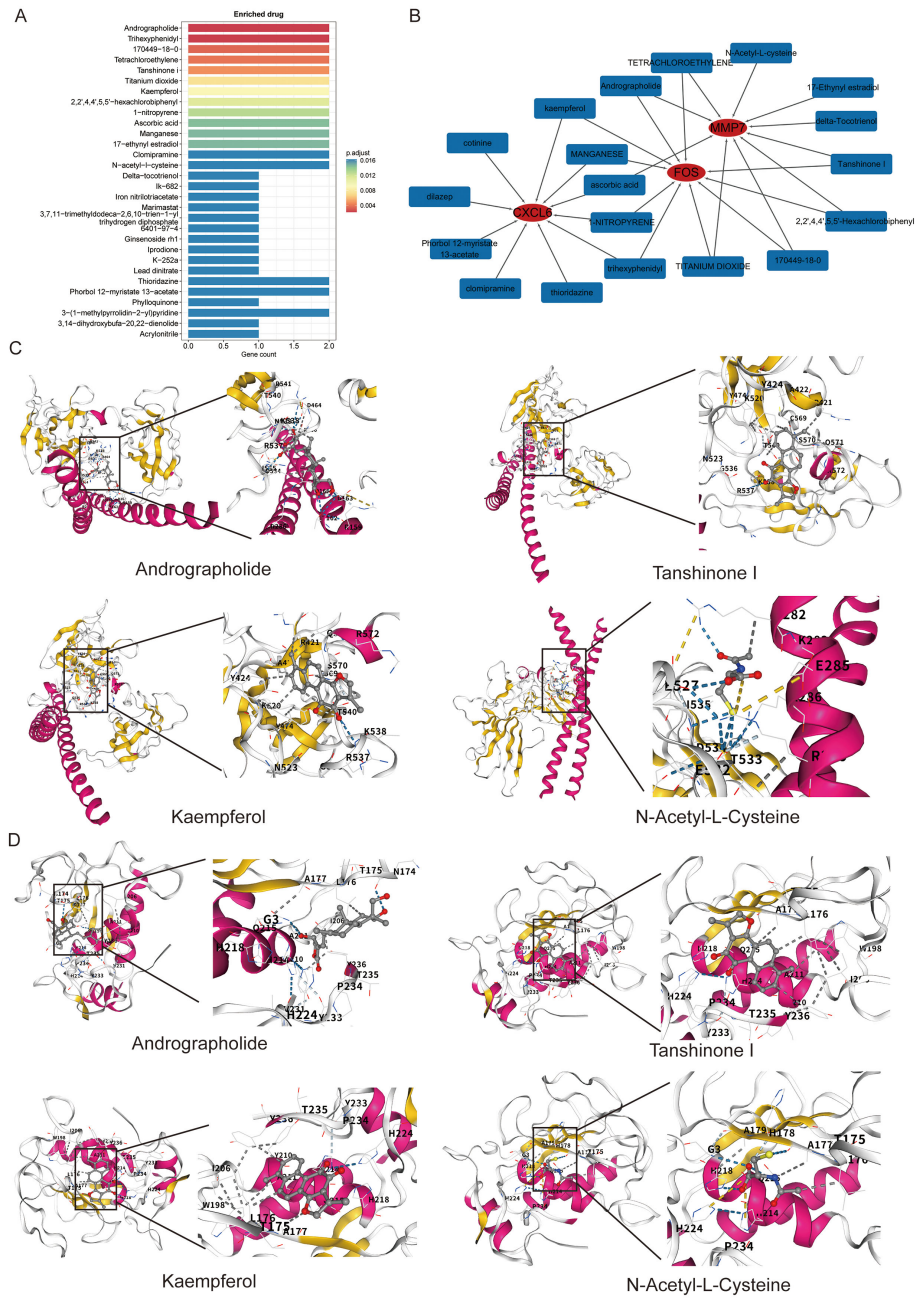
Screening of potential therapeutic compounds targeting the NETs signature. **(A)** Bar plot showing results of drug enrichment analysis from the DSigDB database. **(B)** A drug-target interaction network linking candidate natural compounds with key genes from the signature. **(C, D)** Predicted binding modes from molecular docking simulations, showing interactions between selected compounds and the binding pockets of FOS **(C)** and MMP7 **(D)**.

Finally, to explore the therapeutic tractability of the identified signature, we performed drug-gene interaction analysis using the DSigDB database. This screen identified several candidate compounds with known anti-inflammatory or antioxidant properties, including andrographolide, kaempferol, tanshinone I, and NAC, that were significantly associated with FOS, MMP7, and CXCL6 (**Figures 7A, B**). To further assess these candidates, molecular docking was performed. The analysis predicted stable binding of these compounds to the active sites of both FOS and MMP7, characterized by favorable binding affinities and the formation of multiple hydrogen bonds and hydrophobic interactions with key residues (**Figures 7C, D**). These computational findings support the potential of these compounds to modulate the activity of our signature genes and provide a strong rationale for future preclinical testing.

## 4 Discussion

ASH is an aggressive form of ALD characterized by profound neutrophilic infiltration, severe hepatocellular injury, and a dismal prognosis (35). Current therapies, such as corticosteroids, pentoxifylline, and NAC, primarily function by blunting downstream inflammatory or oxidative cascades rather than the specific upstream immune drivers of ASH, thereby limiting their long-term survival benefits (36). In this context, NETs have emerged as key contributors to alcohol−induced liver damage, as shown by increased NET formation in patients and animal models, the protective effect of neutrophil depletion, and the benefit of recombinant G−CSF in experimental settings (11). However, the core transcriptomic signature orchestrating this NET-driven inflammatory cascade remains obscure. Our study addresses this gap by identifying a robust three-gene signature (FOS, MMP7, and CXCL6) that serves as a central hub for NET-driven inflammation. We demonstrate that this signature not only exhibits high diagnostic accuracy across cohorts but also captures the dynamic, stage-specific evolution of ASH, stratifying patients into distinct metabolic and immune subtypes that may guide precision therapy.

Within this signature, FOS exhibits distinctive dynamic behavior. In our study, FOS was transiently induced in the short−term alcohol exposure phase but became markedly suppressed in the long−term ASH model and in patient samples. This biphasic regulatory pattern suggests a temporal switch in its function. In the early stages, we postulate that the chaotic ASH microenvironment, characterized by lipid dysregulation, oxidative stress, and inflammatory imbalance, serves as the potent upstream trigger for the initial FOS surge (37). As a canonical immediate−early gene and a key component of the AP−1 transcription factor complex, elevated FOS levels likely integrate extracellular stress signals into inflammatory and regenerative gene expression programs, driving the initial inflammatory burst by amplifying pro-inflammatory mediators (38–40). Concurrently, FOS can bind to the PAD4 promoter, thereby upregulating PAD4 expression and promoting NET formation (41). In contrast, despite the persistence of metabolic and inflammatory stress in chronic disease, FOS expression is markedly suppressed. This paradoxical downregulation may be consistent with stress adaptation, negative feedback, cellular exhaustion, and ultimately regenerative failure (42). Conceptually, this ‘inflammation–regeneration uncoupling’ provides a plausible framework for advanced ASH, in which strong, NET-mediated inflammatory activity persists against a background of exhausted hepatocellular repair.

In contrast to the biphasic trajectory of FOS, MMP7 and CXCL6 demonstrated a persistent upregulation throughout the course of the disease, thereby functioning as the persistent “engines” of chronic pathology. Biologically, CXCL6 acts as a potent CXCR1/2 ligand driving neutrophil recruitment and NET formation (43), while MMP7 regulates chemokine gradients by cleaving the syndecan−1–CXCL1 complex, thereby sharpening chemotactic cues (44, 45). Other studies suggest that MMP7 can directly induce CXCL6 expression, providing a mechanistic link between matrix remodeling and neutrophil chemotaxis (46). We therefore propose that ASH evolves from an early, FOS−driven acute response into a self−sustaining chronic phase dominated by this MMP7–CXCL6 axis. Ultimately, this axis likely orchestrates a broader deterioration of the hepatic ecosystem, where neutrophil−derived proteases and reactive oxygen species damage sinusoidal endothelial cells and activate stellate cells, thereby coupling NET−rich inflammation to progressive fibrosis.

Although alcoholic steatohepatitis (ASH) and non-alcoholic steatohepatitis (NASH) share histological hallmarks—including steatosis, ballooning, and lobular inflammation—our analysis underscores fundamental differences in their NET-related biology. Clinically, NASH manifests as a predominantly ‘proliferative–metabolic’ inflammation driven by chronic lipotoxicity and hyperinsulinemia, with a distinct trajectory toward cirrhosis and hepatocellular carcinoma (47). In this context, FOS is frequently identified as a sustained hub gene linking metabolic reprogramming to IL-17-driven inflammation (48), whereas CXCL6 and MMP7 levels typically parallel fibrosis stages, reflecting cumulative inflammatory-fibrotic remodeling rather than an abrupt event (49, 50).Conversely, ASH is characterized by ‘inflammation–regeneration uncoupling,’ reflecting a sustained, cytokine-driven neutrophil ‘storm’ that predisposes to acute liver failure (12, 51). In our models and patient samples, this pathology is mirrored by a distinct ‘rise-then-fall’ FOS trajectory occurring against a background of persistently elevated MMP7 and CXCL6. This divergence may highlight a critical distinction: unlike the sustained proliferative signaling in NASH, the collapse of FOS in advanced ASH suggests a catastrophic failure of regenerative capacity amidst ongoing neutrophilic injury, providing a molecular rationale for the rapid clinical decompensation characteristic of severe ASH.

From a clinical perspective, the FOS-MMP7-CXCL6 signature offers significant clinical utility. Diagnostically, the model achieved near-perfect discrimination (AUC ≈ 1.0), underscoring its potential as a non-invasive biomarker panel, particularly given that MMP7 and CXCL6 are readily detectable secreted proteins (52). Beyond diagnostics, this signature effectively stratified patients into two biologically distinct subtypes: a ‘Fibro-inflammatory Subtype’ (C2) enriched in cytotoxic immune pathways, and a ‘Metabolic Subtype’ (C1) defined by metabolic signatures. This stratification could enable precise risk assessment and guide tailored interventions. Therapeutically, our drug enrichment analysis and molecular docking identified several candidate anti-inflammatory and antioxidant agents predicted to modulate the NETs-related signature. Among these, the identification of NAC is particularly compelling, given its established clinical benefits in severe alcoholic hepatitis, with greater benefit at 1 month when combined with corticosteroids (53). However, given the indispensable role of neutrophils in host defense, therapeutic strategies targeting NETs or neutrophil trafficking must carefully balance attenuation of pathological inflammation against the risk of infection in this highly vulnerable population.

We acknowledge several limitations in this study. First, despite rigorous bioinformatic correction, inter-cohort variability may persist. Second, while we infer the FOS-NETs link based on expression patterns and literature, we did not experimentally dissect this specific interaction in our models, such as ChIP-seq. Third, bulk transcriptomic profiling limits our ability to definitively assign the gene signature to specific cellular compartments. Future studies should therefore focus on single-cell omics to map the cellular origins of these signals and prospective clinical cohorts to validate the diagnostic utility of the identified subtypes.

## 5 Conclusion

Collectively, our results identify NETs as a central hub in ASH immunopathology, propose the key role of the FOS-MMP7-CXCL6 core axis in disease progression, and provide an evidence chain for molecular subtyping. Following further validation, this axis has the potential to be translated into actionable diagnostic and therapeutic tools, offering new insights for improving outcomes in ASH patients.

## Data Availability

The original contributions presented in the study are included in the article/**Supplementary Material**. Further inquiries can be directed to the corresponding authors.
