# Supplementary material for: A stage specific NETs-related signature in alcoholic steatohepatitis: from molecular subtyping to therapeutic vulnerabilities
Source: Front Immunol. 2026 Jan 14;16:1711388. doi: 10.3389/fimmu.2025.1711388 (PMC12846975; doi:10.3389/fimmu.2025.1711388)
Supplement: Supplementary file 1 [file DataSheet1.pdf]

A Stage Specific NETs-Related Signature in Alcoholic Steatohepatitis: From Molecular Subtyping to Therapeutic Vulnerabilities

Wei Gao[1,2,3,#], Zhiyong Lin[1,2,3,#], Yuntao Bao[1,2,3], Mingjiang Liu[1,2,3], Guangtao Ma[1,2,3], Xianxiang Chen[1,2,3], Shuiping Yu[1,2,3,*], and Yonglian Zeng[1,2,3,*]

[1]Division of Hepatobiliary Surgery, the First Affiliated Hospital of Guangxi Medical University, Nanning, Guangxi 530021, China.

[2]Key Laboratory of Early Prevention and Treatment for Regional High Frequency Tumor (Guangxi Medical University), Ministry of Education, Nanning, Guangxi 530021, China.

[3]Guangxi Key Laboratory of Immunology and Metabolism for Liver Diseases, Nanning, Guangxi 530021, China.

[#]These authors contributed equally to this work.

***Correspondence：** Yonglian Zeng and Shuiping Yu, Guangxi Key Laboratory of Immunology and Metabolism for Liver Diseases, the First Affiliated Hospital of Guangxi Medical University, NO 6 Shuangyong Road, Nanning, Guangxi 530021, China. E-mail: zyl-lian@163.com and yushuiping03@163.com.

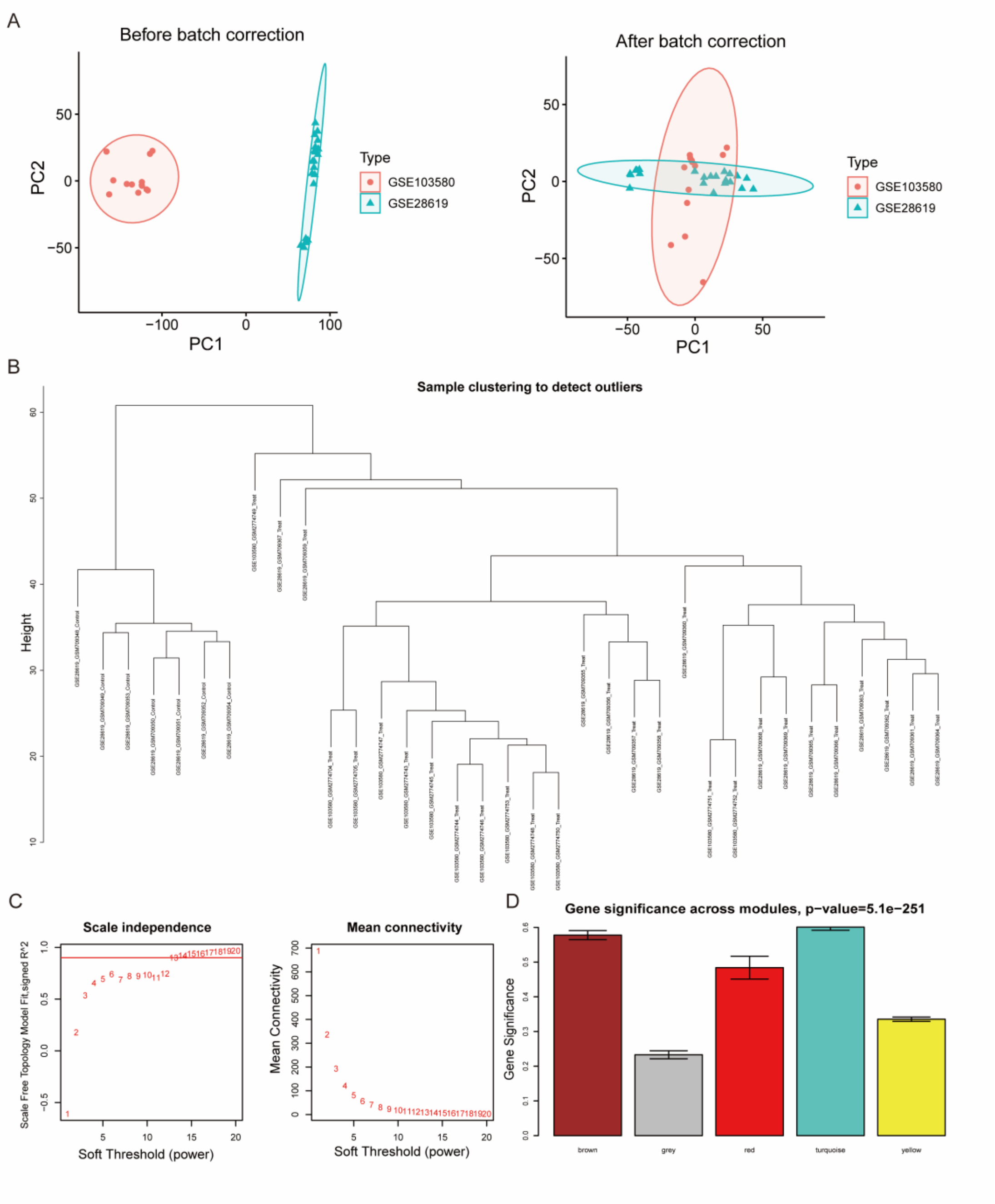

Supplement Figure 1. Data quality control and construction of the weighted gene co-expression network.

(A) Principal Component Analysis plots of samples before and after batch effect correction, demonstrating group separation.

(B) Sample clustering tree diagram used to detect and remove outlier samples.

(C) Analysis of scale-free topology model fit and mean connectivity

(D) Bar plot illustrating gene significance across modules.

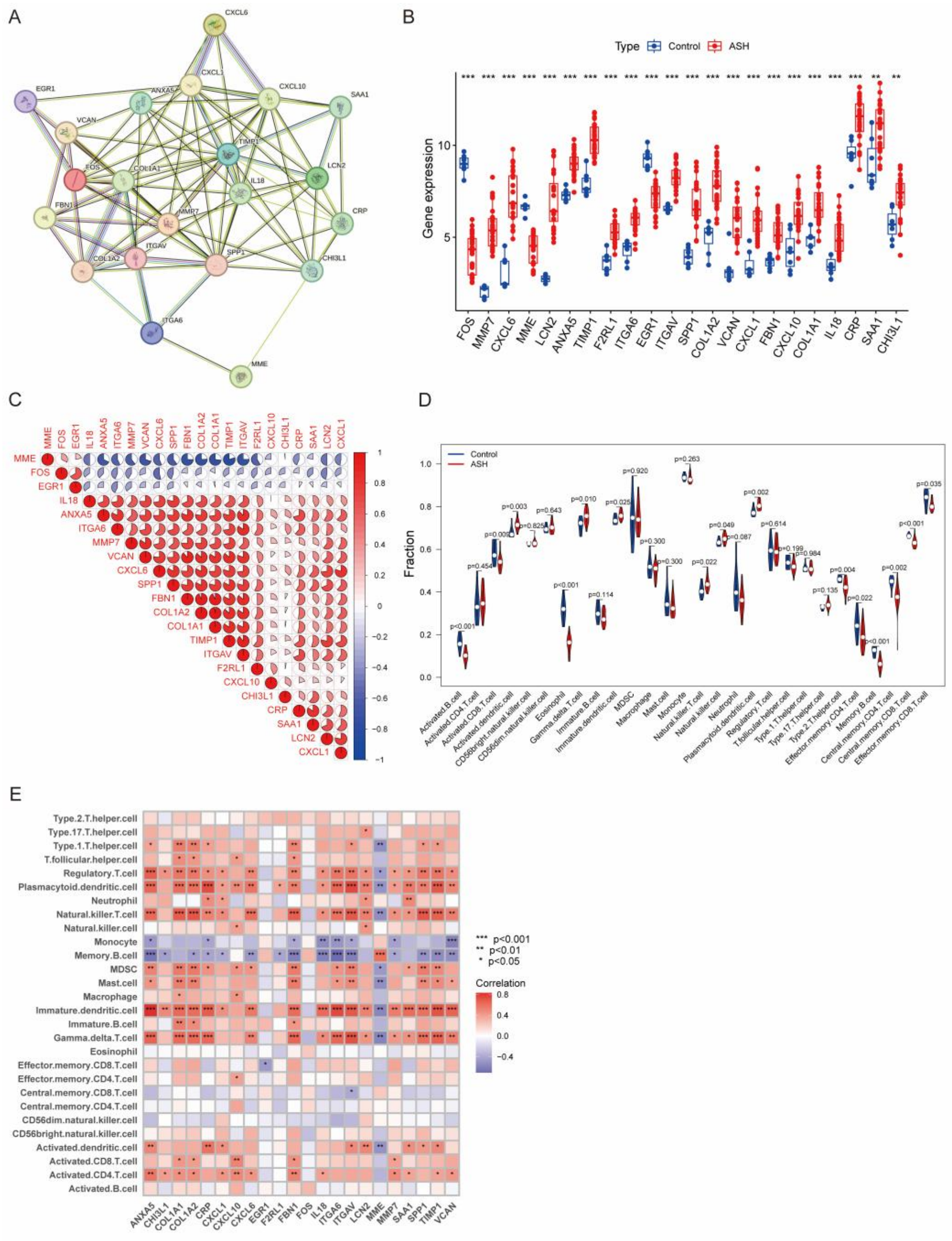

Supplement Figure 2. Analysis of 22 hub gene expression patterns and immune cell infiltration.

(A) Protein-protein interaction (PPI) network of the hub genes, visualizing their functional associations.

(B) Differential expression of the hub genes between the ASH and control groups.

(C) Correlation matrix displaying the co-expression relationships among the 22 hub genes.

(D) Comparison of immune cell infiltration levels between control and ASH samples.

(E) Heatmap illustrating the relationship between the expression levels of 22 hub genes and the infiltration levels of various immune cell subtypes. *$p < 0.05$, **$p < 0.01$, ***$p < 0.001$.

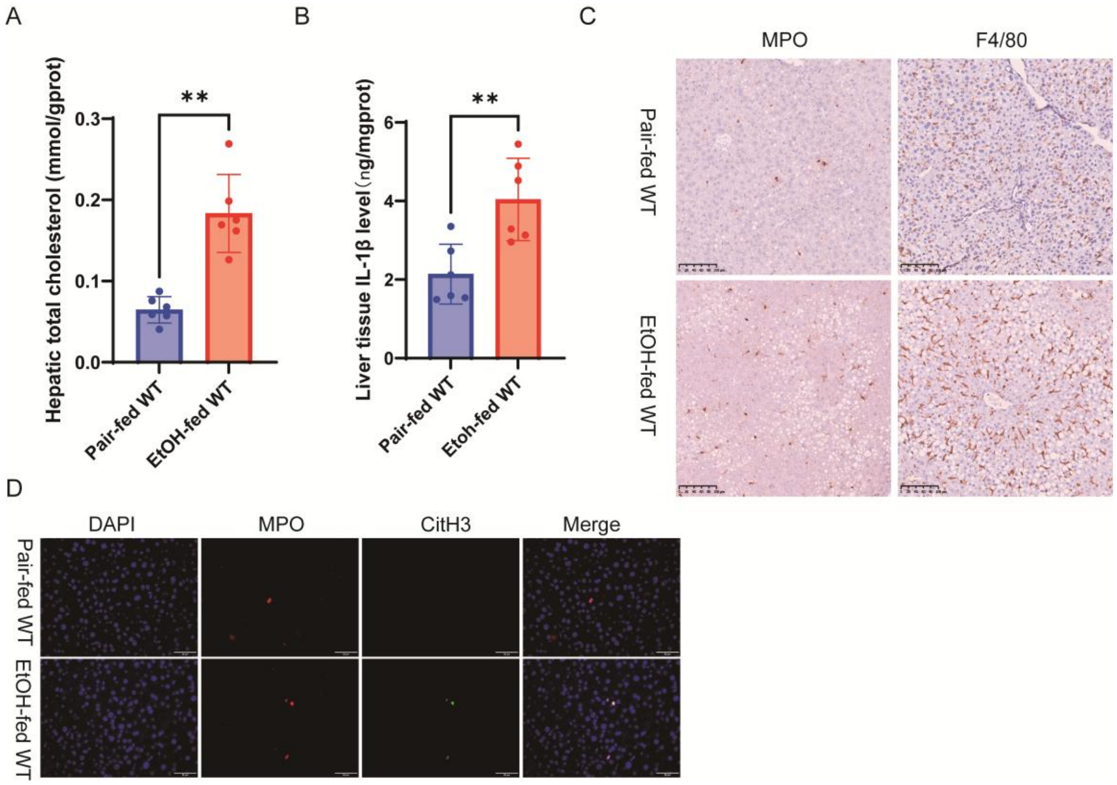

Supplement Figure 3. validation of hepatic injury, inflammation, and NET formation in the short-term NIAAA model.

(A) Hepatic levels of TC.

(B) ELISA quantification of the pro-inflammatory cytokine IL-1β in liver homogenates.

(C) Representative immunohistochemical staining for MPO and F4/80 in liver sections (200× magnification; scale bar = 100 μm), showing increased immune cell infiltration in EtOH-fed mice.

(D) Immunofluorescence co-staining of MPO and CitH3 with DAPI, assessing NETs formation. Scale bar = 50 μm. Data are presented as mean ± SD (*$p < 0.05$, **$p < 0.01$, ***$p < 0.001$).

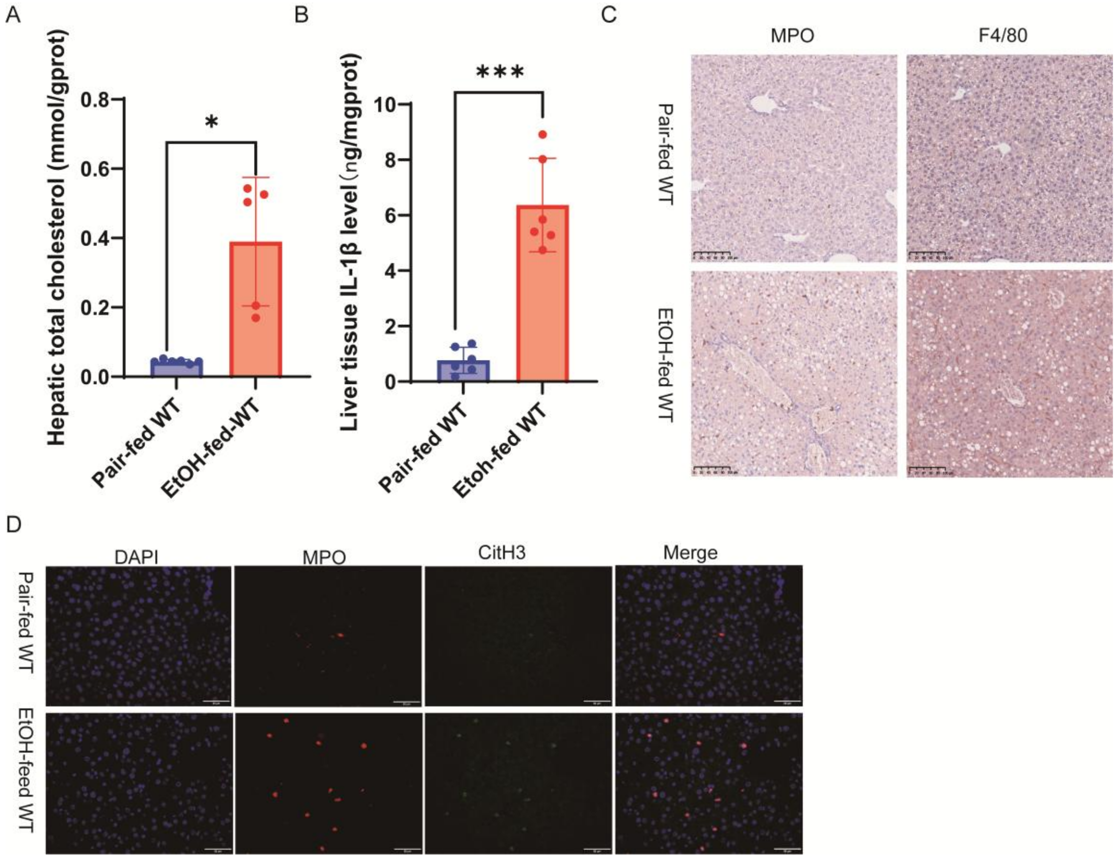

Supplement Figure 4. Validation of hepatic injury, inflammation, and NETs deposition in a Chronic ASH Mouse Model

(A) Quantification of hepatic Total Cholesterol (TC) accumulation.

(B) Assessment of hepatic IL-1β levels by ELISA.

(C) Representative liver histology showing MPO and F4/80 Immunohistochemistry staining (200× magnification; scale bar = 100 μm).

(D) Immunofluorescence co-staining of MPO and CitH3 with DAPI, assessing NETs formation. Scale bar = 50 μm

Data are presented as mean ± SD (* $p < 0.05$, ** $p < 0.01$, *** $p < 0.001$).
